# Supplementary figures and images for: Design principles for the glycoprotein quality control pathway
Source: PLoS Comput Biol. 2021 Feb 1;17(2):e1008654. doi: 10.1371/journal.pcbi.1008654 (PMC7877790; doi:10.1371/journal.pcbi.1008654)

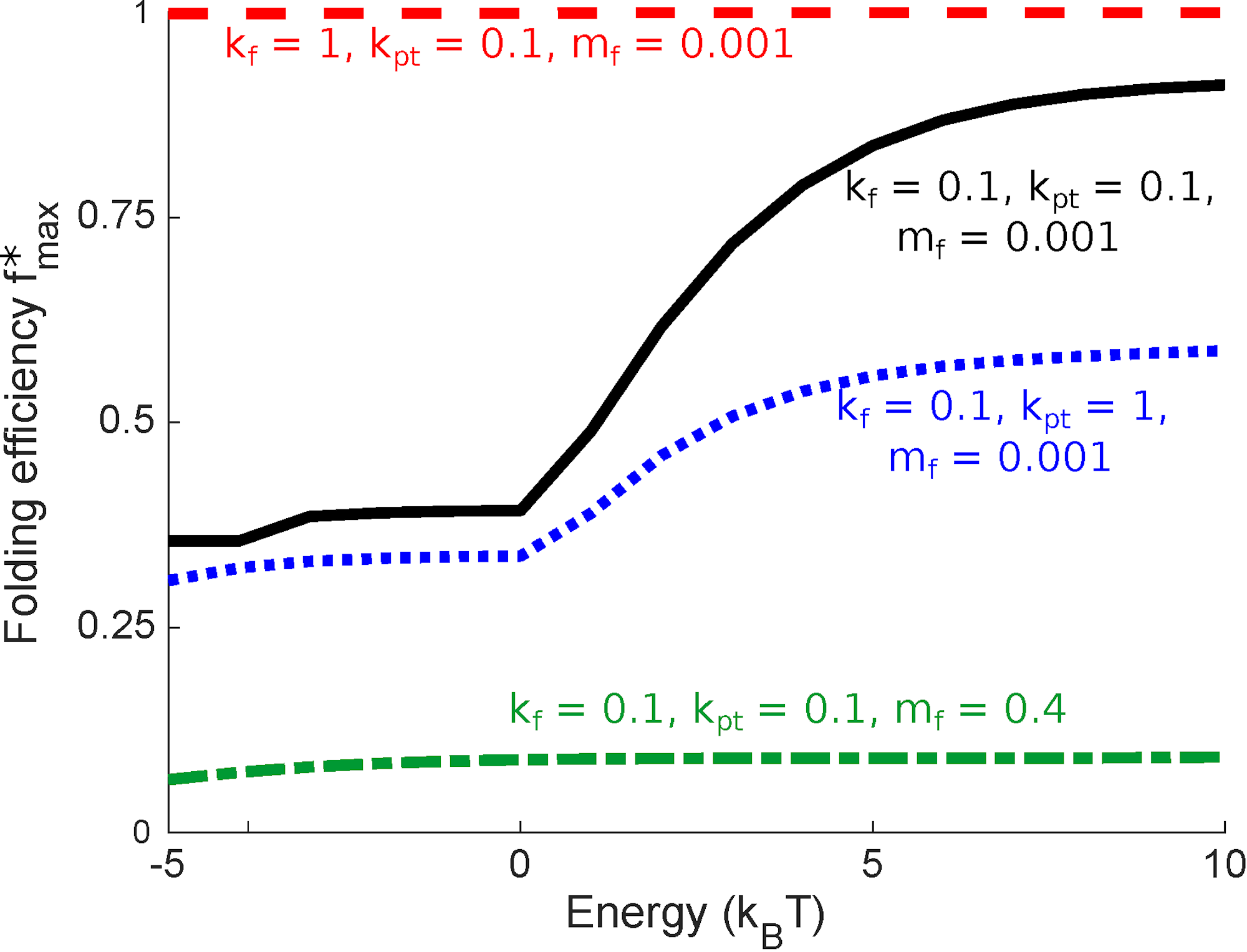

Supplement: S1 Fig — Each curve adjusts kc, k-c, kr, k-r, kg, k-g, and kd to maximize the folding fraction (Eq 2) while the total cycle energy (Eq 3) is varied and the total unfolded protein is constrained to Punfolded=Pg+Pg*+Pc+Pc*+P+P*=1. Other parameters are fixed for each curve as indicated, and background protein levels are set to Pb = 1. Colored curves show effects of increasing protein folding speed (red dashed), increasing protein production (blue dotted), and increasing misfolded fraction (green dashed-dotted) relative to the black curve, which is identical to the corresponding curve in Fig 2B. Increased kf leads to a higher folding efficiency, because faster folding can better compete with degradation, allowing folded proteins to rapidly leave the cycle and free up chaperones. Both high protein production (kpt) and high misfolded fraction (mf) lead to decreased folding efficiency because fewer chaperones are unoccupied and available for foldable protein binding. (TIF) [file pcbi.1008654.s001.tif]

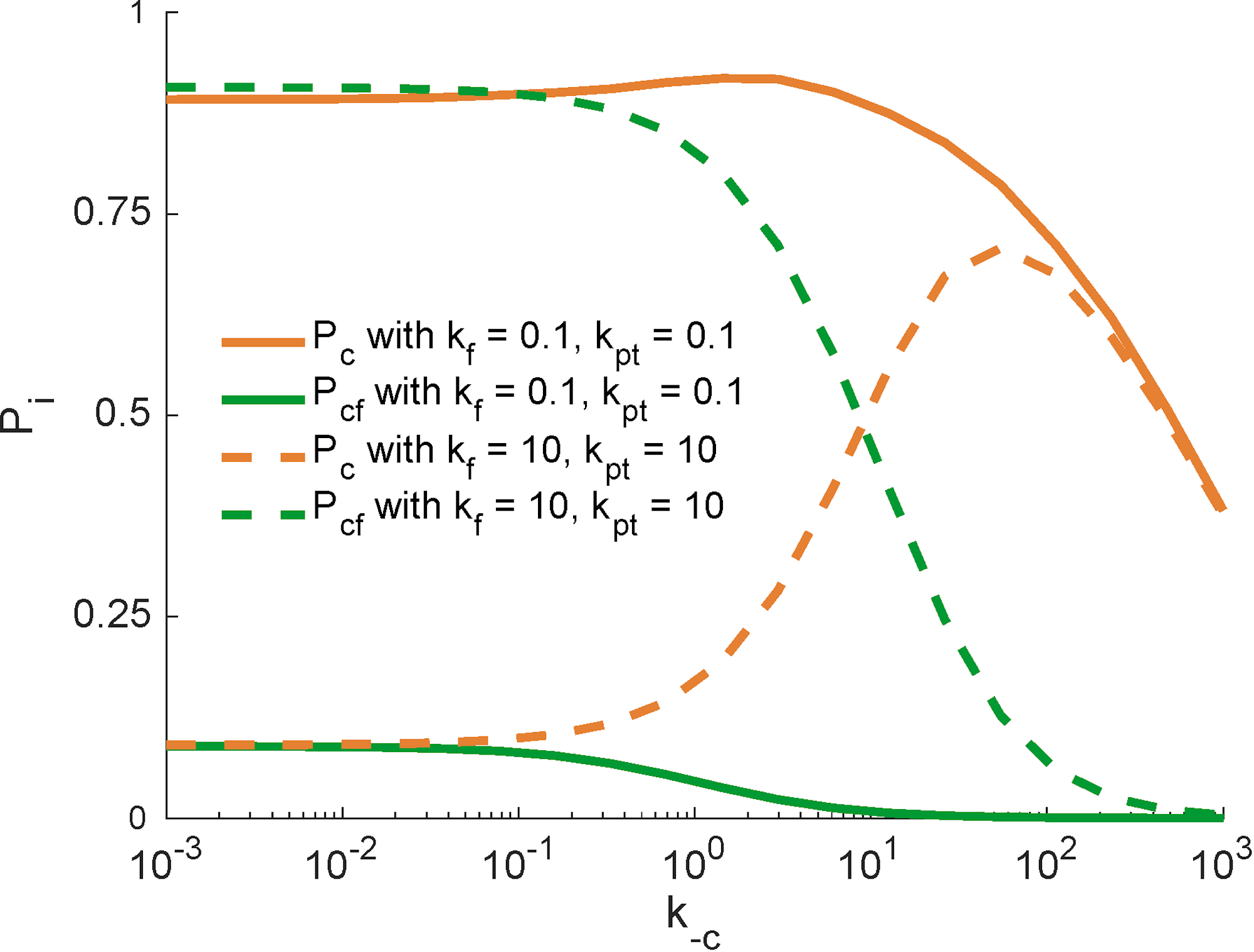

Supplement: S2 Fig — Steady-state concentrations of chaperone-bound foldable proteins (Pc, orange), and already-folded proteins (Pcf, green) vs. unbinding rate k-c. Two regimes are shown, corresponding to two curves shown in Fig 3B. Solid lines correspond to low production, slow folding. Dashed lines show high production, rapid folding. mf = 0.001 for all curves. Increased k-c allows already-folded proteins to be rapidly removed from chaperones, freeing chaperones to bind other nascent proteins. For fast folding, already-folded proteins (Pcf, green dashed line) can occupy a significant fraction of the available chaperones, leading to a decrease in efficiency for low unbinding rates k-c—this occupation of significant chaperone by already unfolded proteins at low k-c is not seen for slow-folding proteins (green solid line). If the unbinding rate becomes much higher than the folding rate, proteins will frequently detach from the chaperone before they can fold, manifesting as low values of Pc (orange lines), with rapid unbinding reducing efficiency at all folding rates. (TIF) [file pcbi.1008654.s002.tif]
